# Supplementary material for: Mpox Person-to-Person Transmission—Where Have We Got So Far? A Systematic Review
Source: Viruses. 2023 Apr 28;15(5):1074. doi: 10.3390/v15051074 (PMC10222665; doi:10.3390/v15051074)
Supplement: Supplementary file 1 [file viruses-15-01074-s001.zip › viruses-2341124-supplementary.pdf]

Mpox Person-to-Person Transmission—Where Have We Got So Far? A Systematic Review

Pedro Pinto [1], Miguel Alves Costa [2], Micael F. M. Gonçalves [1], Acácio Gonçalves Rodrigues [1,3] and Carmen Lisboa [1,3,4,*]

[1] Division of Microbiology, Department of Pathology, Faculty of Medicine, University of Porto, 4200-319 Porto, Portugal
[2] Department of Dermatology and Venereology, Centro Hospitalar Vila Nova de Gaia/Espinho, 4434-502, Porto, Portugal
[3] Centre for Health Technology and Services Research/Rede de Investigação em Saúde (CINTESIS/RISE), Faculty of Medicine, University of Porto, 4200-319 Porto, Portugal
[4] Department of Dermatology and Venereology, University Hospital Centre of São João, 4200-319 Porto, Portugal
* Correspondence: carlis@med.up.pt

Supplementary Table S1. Queries used for database search in PubMed, Scopus and Web of Science.

**PubMed**	Monkeypox[Title/Abstract] AND (transmission[Title/Abstract] OR transmissions[Title/Abstract] OR transmissibility[Title/Abstract] OR transmissible[Title/Abstract] OR clothing[Title/Abstract] OR clothes[Title/Abstract] OR contact[Title/Abstract] OR surface[Title/Abstract] OR surfaces[Title/Abstract] OR fomite[Title/Abstract] OR fomites[Title/Abstract] OR particle[Title/Abstract] OR particles[Title/Abstract] OR sexual[Title/Abstract] OR mucous[Title/Abstract] OR contaminated[Title/Abstract] OR contamination[Title/Abstract] OR respiratory[Title/Abstract] OR skin[Title/Abstract] OR skin-to-skin[Title/Abstract]) AND (humans[All Fields] OR human[All Fields]) NOT (review[Publication Type]) NOT (editorial[Publication Type]) NOT (case reports[Publication Type])
**Scopus**	(TITLE-ABS-KEY (transmission OR transmissions OR transmissibility OR transmissible OR clothing OR clothes OR contact OR surface OR surfaces OR fomite OR fomites OR particle OR particles OR sexual OR mucous OR contaminated OR contamination OR respiratory OR skin OR skin-to-skin) AND TITLE-ABS-KEY (monkeypox) AND ALL (human OR humans)) AND (EXCLUDE (DOCTYPE , "re") OR EXCLUDE (DOCTYPE , "ed") OR EXCLUDE (DOCTYPE , "no") OR EXCLUDE (DOCTYPE , "ch"))
**Web of Science**	AB=(Monkeypox) AND AB=(transmissibility OR transmissible OR transmission OR transmissions OR fomite OR fomites OR sexual OR surface OR surfaces OR clothing OR clothes OR mucous OR skin OR skin-to-skin OR contact OR contamination OR contaminated OR respiratory OR particle OR particles) NOT (Book Review OR Database Review OR Editorial Material OR Review (Document Type)) AND (humans OR human (All Fields))
